# Perception of Official Corruption, Satisfaction With Government Performance, and Subjective Wellbeing—An Empirical Study From China

**DOI:** 10.3389/fpsyg.2022.748704

**Published:** 2022-07-28

**Authors:** Jiazheng Ma, Bin Guo, Yanghang Yu

**Affiliations:** ^1^Shanghai Administration Institute, Shanghai, China; ^2^School of Public Administration, Northwest University, Shaanxi, China; ^3^School of Public Finance and Management, Yunnan University of Finance and Economics, Yunnan, China

**Keywords:** perception of official corruption, satisfaction with government performance, subjective wellbeing, mediating effect, China

## Abstract

Both corruption and subjective wellbeing are of concern to academics and governments. Although some evidence suggests that corruption deteriorates subjective wellbeing, the relationship between perception of official corruption and subjective wellbeing is still unknown. This study aims to examine the link between perceived official corruption and subjective wellbeing in the context of China and whether satisfaction with government performance has a mediating effect in the process. Based on data from China General Social Survey, a structural equation model was used to test the hypotheses. The results of 3,033 Chinese respondents suggest that perception of official corruption is negatively related to subjective wellbeing, and satisfaction with government performance plays a mediating role in the relationship between perception of official corruption and subjective wellbeing.

## Introduction

Governments have become the strongest organizations in our society because they possess a large amount of resources. Scholars have recognized government can have great impacts on people’s happiness, including government quality (Helliwell and Huang, 2008; Ott, 2018), government spending (Chen et al., 2016; Flavin, 2019), government ideology (Bjørnskov et al., 2007; Dreher and Öhler, 2011), and government size (Ott, 2015; Sequeira et al., 2017). Given the economic hypothesis that every individual, as a rational decision-maker, seeks to maximize their own interests, there is a perception that government officials tend to be corrupt when corruption benefits outweigh corruption costs (Ni, 2009). Previous studies about corruption and wellbeing have focused on the personal experience of corruption (Singer, 2013; Wu and Zhu, 2016) or taken corruption as one element of the quality of government (Tavits, 2008; Ott, 2010), however, the perspective of official corruption has been overlooked. Official corruption has some negative consequences—it can exacerbate income inequality and poverty (Justesen and Bjørnskov, 2014), damage economic productivity (Johnson et al., 1997), and waste public resources (Liu and Mikesell, 2014)—but the effects of official corruption on individuals’ wellbeing have not yet been uncovered.

Public administration has been concerned with government performance since its inception, one important mission for the government’s administrative machinery is how to achieving high performance (Coggburn and Schneider, 2010). Administrative activities that take place within government have a direct influence on the outputs and outcomes of the public agencies. Management methods, bureaucratic structure, and official behaviors have great impact on government performance (Thomas, 2005; Coggburn and Schneider, 2010; Mac Carthaigh et al., 2016). Researches focused on how to improve government programs and services through performance evaluation (Ryzin, 2015; Mullin, 2021; Park et al., 2021), and, ultimately, the status of government in citizens’ eyes (Lynn et al., 2000; Tran and Dollery, 2021).

Citizens are the consumers of the public service supplied by the government, so their satisfaction should be the ultimate internal evaluation of governance. The more satisfied the public with government performance, the easier it is for the government to implement policies, and satisfaction with government performance can maintain citizens’ political trust when the policy fails (Saich, 2006). Waldo (1955) has pointed out that the welfare, happiness, and very lives of all of us depend in large measure upon the performance of the administrative mechanisms that surround and support us. Although Jerrell and Saundra (2003) and Whiteley et al. (2010) both investigated the direct relationship between government performance and happiness, they neglected to evaluate the government performance from a citizen perspective (Wang, 2010). Not much is known with regard to how satisfaction with government performance is related to citizens’ wellbeing.

Citizens’ satisfaction with the government may exhibit specific patterns in accordance with country’s administrative frameworks (Huang, 2018). China has experienced eight waves of administrative reforms from 1982 to 2018 to improve government performance, and previous studies believe that those reforms are mostly domestically based and have “Chinese characteristics” (Gao, 2010; Zang and Wang, 2018). In addition, the aim of the Chinese government is to serve the people, and so citizens’ wellbeing should be officials’ goal. Even there are very few studies discussing the effect of Chinese official corruption on regional economic development (Tu et al., 2018; Huang et al., 2019), the relationship between official corruption and citizens’ wellbeing in China is still unknown.

To fill these gaps, we used the data from China General Social Survey (CGSS) (2015) to examine the impact of perception of official corruption on subjective wellbeing and the mediating role of satisfaction with government performance. The government needs officials to maintain its normal operation, and the quality of government officials determines the management capacity and government performance. Since the economic reform launched in 1978, China has witnessed rapid economic growth and a general improvement of social welfare. However, corruption has become an increasingly serious problem for the country (Feng et al., 2018). The results will contribute to extend the study of corruption, government performance, and happiness in developing countries; it will also be useful for governments to reduce official corruption behaviors, improving government performance, and increasing citizens’ subjective wellbeing.

## Literature Review and Hypothesis

### Perception of Official Corruption and Subjective Wellbeing

Subjective wellbeing refers to a broad psychological phenomenon, including both emotional and cognitive elements (Diener, 1984; Diener et al., 1999), it is not only the eternal pursuit of individual but also the goal of public administration (Ott, 2018; Fan et al., 2022). Reliable and valid subjective wellbeing can be interpreted as an important factor of formulation of public policy (Cummins, 2018). Good governance will foster a sense of fairness and trust, which are known as contributors to enhanced wellbeing (Helliwell et al., 2017). The degree of corruption is one of the most important indicators of good governance, which is regarded as a negative predictor of wellbeing.

The literature about the corruption and happiness largely illustrates these entities on a macro level or micro level. On the macro level, corruption is recognized as an important indicator of government quality, and many scholars have found that the quality of government is significantly related to happiness (Tavits, 2008; Bjørnskov et al., 2010; Ott, 2010). Citizens living in countries where corruption is less common are relatively more satisfied with their lives than those living in countries where corruption is widespread (Helliwell, 2003; Kim and Kim, 2012). Helliwell and Huang (2008) and Teorell (2009) provide additional evidence on the positive effect of good governance on happiness. Welsch (2008) finds that subjective wellbeing is affected by corruption indirectly through GDP, and also directly through non-material factors. In addition, corruption also undercuts democratic political processes, negatively affecting citizens’ subjective wellbeing (Tavits, 2008).

On the micro level, personal experience of corruption can influence their happiness. Singer (2013) finds that bribery undermines victims’ individual subjective wellbeing. Experienced corruption has a detrimental effect on individuals’ mental health (Gillanders, 2011), and being involved in corrupt exchanges makes people unhappy (Chrikov and Ryan, 2001). Bribery (both bribing and being bribed) can negatively influence happiness, because people may feel guilt and displeasure about violating the law (Wu and Zhu, 2016). Also, Sulemana (2014) finds that fear of crime is negatively related to happiness. Previous studies suggest that corruption affects subjective wellbeing through the perspective of quality of government and personal experience, but the effects and mechanisms of official corruption on citizens’ wellbeing remain ambiguous.

Corruption is defined as the “misuse of public office for private gain” (Sandholtz and Koetzele, 2000, p. 32). From this perspective, public officials always make unsuitable public policies for their private interests and selfish goals (Jain, 2001). In addition, official corruption has become a major cause of public dissatisfaction and unhappiness (Brockmann et al., 2009). On the one hand, official corruption makes individuals fell a sense of unfairness and inequality. According to the distributive and procedural justice theory, people evaluate the outcomes based on the quality of distributions and procedures, distributive and procedural justice are associated with satisfaction and wellbeing (Tang and Baldwin, 1996; Lucas et al., 2011; Ng et al., 2020), individuals who perceived injustices of corruption would have lower wellbeing. Corrupt officials may distort the public resource allocations: they are likely to spend more on those who can provide larger benefits to them (Liu and Mikesell, 2014), and there will therefore be less money to spend on public goods which are necessary for ordinary people. With the unfair distribution of society’s resources, citizens believe that they are treated unfairly, and their perception of unfairness and inequality will increase, which will be harmful to their satisfaction (Magalhães, 2016). On the other hand, government officials’ corruption will increase citizens’ distrust in the political system over time, because corrupt officials take advantage of their power for their personal interests rather than the public interest. Political trust is a determinant of subjective wellbeing (Fu, 2017), so officials’ corruption will affect individuals’ happiness negatively. Based on the above, we hypothesize:

*Hypothesis* 1: The perception of official corruption is negatively related to subjective wellbeing.

### Satisfaction With Government Performance and Subjective Wellbeing

Since the late 1980s, government performance has become an important issue of both academic interest and policy significance. International organizations have proposed different conceptualizations of good governance. The United Nations defines good governance as “the process of decision-making and the process by which decisions are implemented (or not implemented)” (United Nations, 2006). The World Bank started the Worldwide Governance Indicators (WGI) project, believing a six-dimensional definition of good governance, including voice and accountability, rule of law, government effectiveness, political stability and absence of violence, regulatory quality, and control of corruption (see Kaufmann et al., 1999). Although the indicators faced several challenges (Bratton and Chang, 2006), good governance should be operated as executable policy tools to achieve sustainable economic, social, and human development (Kaufmann et al., 2008). Citizen satisfaction therefore becomes a widespread performance information metric (Bouckaert et al., 2005) which can help to overcome the difficulties of measuring actual government outcomes (Holzer and Yang, 2004). Citizen satisfaction reflects people’s judgment on the performance of the government and its officials (Ryzin, 2004). Government performance (Adang and Borm, 2007), victimization (Circo et al., 2019), transparency (Yang, 2018), red tape (Tummers et al., 2016), bureaucratic personnel quality (Dahlström et al., 2018), and communication (Ho and Cho, 2016) can influence citizens’ satisfaction. However, the relationship between satisfaction with government performance and subjective wellbeing is as yet uncovered.

Subjective wellbeing refers to people’s cognitive and affective evaluations of their lives, comprising life satisfaction, pleasant affect, and unpleasant affect (Diener, 1984). It is used to describe the level of satisfaction people experience according to their subjective evaluation of their objective living conditions. Happiness is not only the pursuit of individuals, but is also the goal of the public policy. Many nations and organizations have created national accounts of wellbeing to reflect the quality of life. The United Kingdom has assessed subjective wellbeing as input to policy since 2010. The Organization for Economic Cooperation and Development (OECD) issued guidelines on measuring national subjective wellbeing in 2013 (OECD, 2013). In 2012, the leader of China, Xi Jinping, proposed the “China Dream,” being a dream of state prosperity, national rejuvenation, and people’s happiness. Subjective wellbeing not only helps guide decision-makers on policies and actions, but also reflects the government’s performance.

Government performance can influence individuals’ happiness through public policy outcomes directly and indirectly. Firstly, the government makes direct contributions to improve individuals’ happiness by providing high-quality public services. Public services have a fundamental impact on quality of life (Glaser, 1991). Governments have the ability to influence areas, such as public education, public transportation, health, and environmental protection, which are all closely and directly related to citizens’ daily lives. Secondly, the government influences individuals’ happiness indirectly by offering inducements for private behaviors. Government intervention in the economy and society positively influences life satisfaction (Pacek and Radcliff, 2008; Whiteley et al., 2010; Helliwell et al., 2018). For example, when the government implements policies of tax reduction, residents will spend more money to improve their happiness. Bottom-up spillover theory believes satisfaction with all of life’s domains and subdomains has spillover effects on overall quality of life (Andrews and Withey, 1976; Sirgy et al., 2008). The greater the satisfaction with life’s different domains, the greater the subjective wellbeing (Sirgy et al., 2010). Based on the above, we hypothesize:

*Hypothesis* 2: Satisfaction with government performance is positively related to subjective wellbeing.

### The Mediating Effect of Satisfaction With Government Performance

Anderson and Tverdova (2003, p. 104) conclude that “corruption is likely to be an important component of government performance people use to judge,” so it is reasonable to think that perception of official corruption influences satisfaction with government performance. Corruption is the abuse of power by public officials for their private interests or selfish goals; it means the violation of the rules or ethics of public service. As a result, corruption has a large number of negative consequences: it can increase distrust in the government (Anderson and Tverdova, 2003; Zhang et al., 2019), reduce the strength of national climate policies (Rafaty, 2018), and do harm to sustainable economic development (Sharma and Mitra, 2019). All these negative factors disappoint citizens and erode public respect for the government, thus fostering dissatisfaction with government. In addition, there are several empirical studies that directly analyze the negative effects of corruption on citizen satisfaction (Park and Blenkinsopp, 2011; Jonck and Swanepoel, 2016; Saich, 2016; Pellegata and Memoli, 2018). Up to this point, we have hypothesized that satisfaction with government performance will be positively related to subjective wellbeing. We also hypothesized that perception of official corruption will be negatively related to satisfaction with government performance. Taken together, we hypothesize:

*Hypothesis* 3: Perception of official corruption is negatively related to satisfaction with government performance.

*Hypothesis* 4: Satisfaction with government performance plays a mediating role in the relationship between perception of official corruption and subjective wellbeing.

## Materials and Methods

### Data

Data used in this paper were collected through the China General Social Survey (CGSS) (2015). The CGSS, first launched in 2003, was the first nationwide and comprehensive large-scale social survey project in China. CGSS aims to systematically monitor the changing relationship between social structure and quality of life in both urban and rural China. CGSS 2015 is designed and carried out by Renmin University of China (RUC), and a total of 25 different universities and academic institutions participate in the field survey. The data yielded a total of 10,968 face-to-face interview with Chinese residents from 478 communities in 28 provinces (autonomous regions, municipalities) in mainland China. After processing the missing data, there were 3,033 valid data entries.

Of the 3,033 respondents, 1,445 (47.6% of the total) were male, and 1,588 (52.4%) female. A total of 1,073 (35.4%) had only lower education (primary school), 1,425 (47%) had finished junior high school or senior high school, 497 (16.4%) had a Bachelor’s degree, and 38 (1.3%) had a Master’s degree or doctorate. A total of 728 (24%) were unmarried, while 2,305 (76%) were married. In terms of politics, 2,525 (83.3%) were not a member of the Communist Party of China or Communist Youth League, while 508 (16.7%) were. Among the respondents, 2,708 (89.3%) were irreligious, and 325 (10.7%) were religious (Table 1).

**Table 1:** Descriptive statistics.

Variables	Mean	SD	Min	Max
Control variables				
Gender	0.52	0.50	0	1
Education	1.84	0.74	1	4
Age	49.48	17.13	18	94
Hukou	0.41	0.49	0	1
Personal annual income (RMB, after tax)	29,290.45	62,300.10	0	2,000,000
Family economic status	2.67	0.69	1	5
Religion	0.11	0.31	0	1
Health	3.66	1.07	1	5
Marital status	0.76	0.43	0	1
Political affiliation	0.17	0.37	0	1
Housing area (m^2^)	114.38	85.73	7	1,300
Dependent variable				
Subjective wellbeing	3.90	0.81	1	5
Independent variable				
Perception of corruption	2.65	0.82	1	5
Local governors	2.80	0.97	1	5
Policemen	2.70	0.92	1	5
Judges	2.56	0.87	1	5
Procurators	2.53	0.87	1	5
Mediator variable				
Satisfaction with government	3.44	0.64	1	5
Providing medical care	3.37	0.89	1	5
Providing living security	3.45	0.87	1	5
Providing basic education	3.55	0.84	1	5
Defending national security	3.84	0.75	1	5
Combating crimes	3.61	0.81	1	5
Enforcing law fairly	3.33	0.89	1	5
Handling affairs impartially	3.24	0.92	1	5
Environmental protection	3.30	0.91	1	5
Maintain social equity	3.28	0.92	1	5

*N = 3,033; Gender: 0 = male,1 = female; Hukou: 0 = urban,1 = rural; Education: 1 = primary school, 2 = junior high school or senior high school, 3 = Bachelor’s degree, 4 = Master’s degree or doctorate; Family economic status: 1 = far below the regional average, 2 = below the regional average, 3 = the regional average, 4 = above the regional average, 5 = well above the regional average; Marital status: 0 = unmarried, 1 = married; Health: 1 = very unhealthy, 2 = quite unhealthy, 3 = generally health, 4 = quite healthy, 5 = very healthy; Political status: 0 = not a member of the Communist Party of China or Communist Youth League, 1 = member of the Communist Party of China or Communist Youth League; and Religion: 0 = no religious belief, 1 = religious belief*.

### Measures

#### Subjective Wellbeing

Subjective wellbeing was measured by a single item asking residents “All things considered, do you feel happy in your life?.” The response categories were (1) “not happy at all,” (2) “not happy to a certain extent,” (3) “between unhappy and happy,” (4) “happy to a certain extent,” and (5) “very happy.” Self-report measures of subjective wellbeing show adequate validity, reliability, factor invariance, and sensitivity to change (Diener, 1994). Among the respondents 2,404 (79.2%) reported that their life was “happy to a certain extent” or “very happy,” 202 (6.7%) reported that their life was “not happy at all” or “not happy to a certain extent,” and 427 (14.1%) reported that their life was “between unhappy and happy.”

#### Perception of Official Corruption

We measured the perception of official corruption by asking respondents to evaluate the corruption of government officials, including (a) local governors, (b) policemen, (c) judges, and (d) procurators. The respondents indicated the extent to which they perceived these official to be corrupt on a scale from 1 (very incorrupt) to 5 (very corrupt). Higher scores indicate higher perceived official corruption. The composite reliability (CR) is 0.944, and average variance extracted (AVE) is 0.807. *Cronbach’s α* = 0.917, Kaiser–Meyer–Olkin (KMO) and Bartlett’s tests resulted in scores of 0.801 and *χ*^2^ = 10,082.839 (*p* = 0.000).

#### Satisfaction With Government Performance

We assessed the satisfaction with government performance by asking respondents “Are you satisfied with the performance of the government?” including (a) providing medical care, (b) providing adequate living security for the elderly, (c) providing quality basic education, (d) defending national security, (e) combating crimes, (f) enforcing law fairly, (g) handle affairs impartially, (h) environmental protection, and (i) maintain social equity. Participants indicated their satisfaction with the government performance on a five-point Likert scale (1 = very dissatisfied and 5 = very satisfied). Higher scores indicate higher satisfaction with the government performance. The composite reliability (CR) is 0.916, and average variance extracted (AVE) is 0.548. *Cronbach’s α* = 0.897, Kaiser–Meyer–Olkin (KMO) and Bartlett’s tests resulted in scores of 0.926 and *χ^2^* = 12567.087 (*p* = 0.000).

## Results

Prior to hypothesis testing, a one-way ANOVA was run on perception of official corruption, satisfaction with government performance, and subjective wellbeing to assess potential age and education differences. Respondents aged 20–39 reported the highest perceived official corruption (2.803 ± 0.835) and the lowest satisfaction with government performance (3.271 ± 0.649), while respondents aged above 60 reported the lowest perceived official corruption (2.522 ± 0.788) and the highest satisfaction with government performance (3.561 ± 0.619). Respondents’ satisfaction with government performance decreased with increasing education level, while as education level increases, perceived official corruption and subjective wellbeing also increase (Tables 2, 3).

**Table 2:** The differences of variables between different groups of age.

	Under 19		20–39		40–59		Above 60			
	*M*	SD	*M*	SD	*M*	SD	*M*	SD	*F*	*p*
Perception of official corruption	2.684	0.623	2.803	0.835	2.638	0.813	2.522	0.788	18.071	0.000
Satisfaction with government performance	3.451	0.567	3.271	0.649	3.463	0.636	3.561	0.619	31.806	0.000
Subjective wellbeing	4.074	0.779	3.944	0.786	3.812	0.821	3.958	0.793	8.258	0.000

**Table 3:** The differences of variables between different educational levels.

	Primary school		High school		Bachelor’s degree		Master’s or doctorate			
	*M*	SD	*M*	SD	*M*	SD	*M*	SD	*F*	*p*
Perception of official corruption	2.552	0.784	2.696	0.818	2.705	0.851	2.879	0.808	8.452	0.000
Satisfaction with government performance	3.594	0.619	3.402	0.637	3.243	0.627	3.083	0.677	43.067	0.000
Subjective wellbeing	3.816	0.868	3.902	0.784	4.048	0.708	4.229	0.598	11.535	0.000

We took gender, education, age, hukou, personal annual income, family economic status, religion, health marital status, political affiliation, and housing area as control variables, and conducted the correlation analysis. According to the results, perception of official corruption, satisfaction with government performance, and subjective wellbeing are significantly related to each other. Of the three variables, the mean of subjective wellbeing (3.899 ± 0.805) is the highest, that of perception of official corruption (2.648 ± 0.815) the lowest. This means that the Chinese respondents were generally happy, while a lower score of perceptions of official corruption means they believed most government officials were not corrupt (Table 4).

**Table 4:** Means, standard deviations, and correlations among variables.

Variables	Mean	SD	1	2	3
1. Perceptions of official corruption	2.648	0.815	1		
2. Satisfaction with government performance	3.441	0.642	−0.351^***^	1	
3. Subjective wellbeing	3.899	0.805	−0.105^***^	0.171^***^	1

***p ≤ 0.001, **p ≤ 0.01, and *p ≤ 0.05.

**Figure 1:**
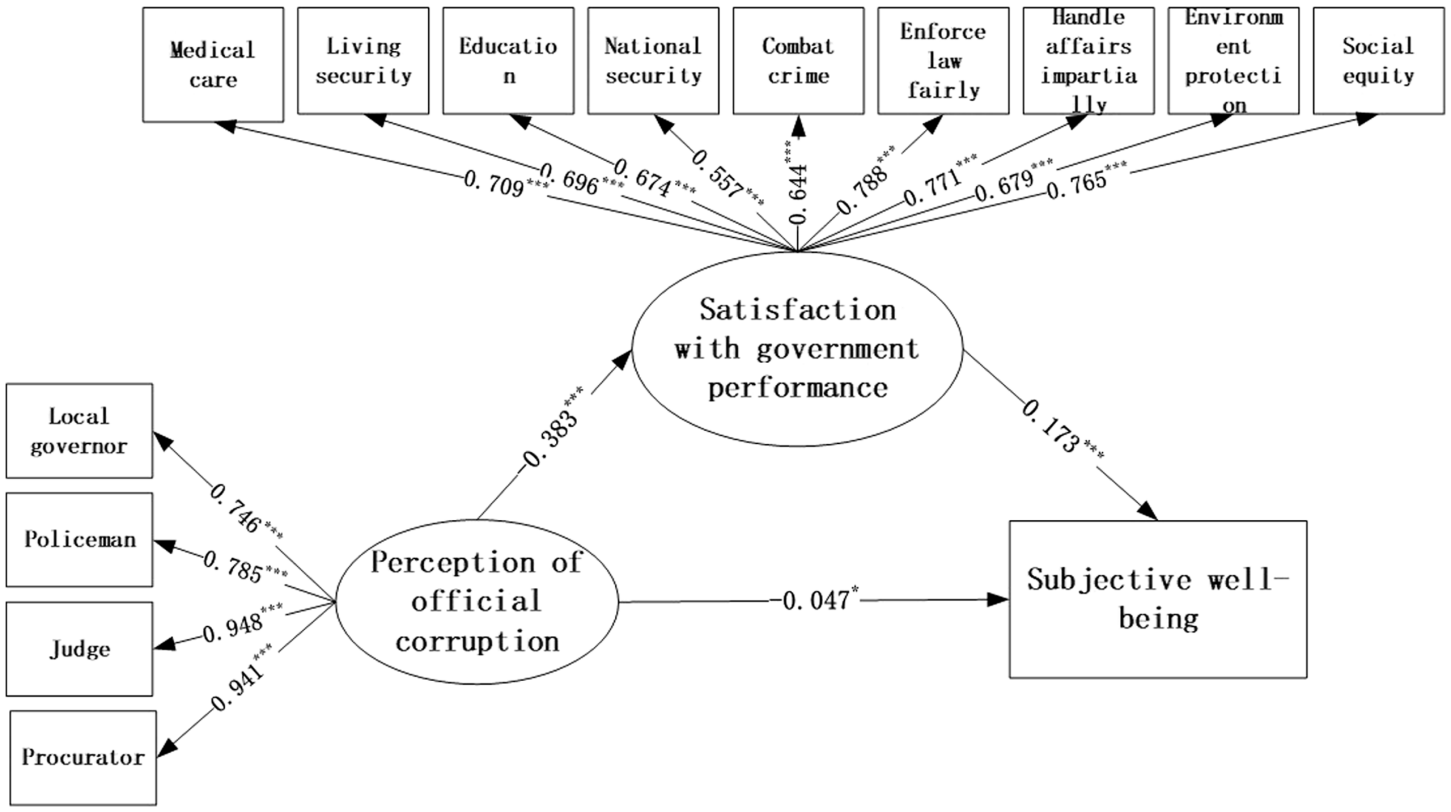
Structural equation modeling results of research model. ***p ≤ 0.001, ***p ≤ 0.01, and *p ≤ 0.05.

To test the hypothesis, structural equation modeling (SEM) analysis was conducted. The model was shown to have good fit indices (CMIN = 732.611, DF = 73, CMIN/DF = 10.036, RMSEA = 0.055, NFI = 0.969, CFI = 0.972, TLI = 0.965, GFI = 0.964, IFI = 0.972; see Figure 1). From Table 5, the perception of official corruption is negatively related to subjective wellbeing (*β* = −0.047, *p* ≤ 0.05), hypothesis 1 is supported. Satisfaction with government performance is positively related to subjective wellbeing (*β* = 0.173, *p* ≤ 0.001), hypothesis 2 is supported. Also, we could find the regression weight “perception of official corruption→ subjective wellbeing” *β* = −0.383 (*p* ≤ 0.001), which means the perception of official corruption is negatively related to satisfaction with government performance, hypothesis 3 is supported.

**Table 5:** Regression weights of the model.

	Estimate	S.E.	C.R.	*p*
Perception of official corruption→ Subjective wellbeing	−0.047	0.022	−2.091	0.032
Satisfaction with government→ Subjective wellbeing	0.173	0.024	7.309	^***^
Perception of official corruption→ Satisfaction with government	−0.383	0.020	−18.976	^***^

***p ≤ 0.001, **p ≤ 0.01, and *p ≤ 0.05.

Bootstrap tests are powerful and can be generalized to mediation analyses when using structural equation modeling methods (Efron and Tibshirani, 1993; Shrout and Bolger, 2002). So, we use a bootstrap sample of 2000 to test the mediating effect of satisfaction with government performance in the relationship between the perception of official corruption and subjective wellbeing. The 95% CIs of the indirect effect is [−0.088, −0.044]. The interval did not overlap with zero. This further indicted satisfaction with government performance mediated the effect of the perception of official corruption on subjective wellbeing, hypothesis 4 was supported.

## Discussion and Conclusion

This research presented in this paper investigates the topic with a sample of Chinese respondents. The results demonstrate that perception of official corruption is negatively related to subjective wellbeing, and satisfaction with government performance plays a mediating role in the relationship between perception of official corruption and subjective wellbeing.

These particular findings have some significant theoretical contributions. Firstly, this study extends our knowledge by providing empirical evidence on the relationship between official corruption and subjective wellbeing. Public officials often distort policies for their private interests, which may reduce residents’ wellbeing. The Corruption Perception Index (CPI) has often been used to measure general perceived corruption of the government in previous studies to discuss the negative relationship between corruption and wellbeing (Tavits, 2008; Singer, 2013; Tay et al., 2014; Amini and Douarin, 2020), while the perceived corruption of different specific occupations of official is often omitted. In addition, the existing literature commonly focuses on the economic consequences of official corruption (Johnson et al., 1997; Justesen and Bjørnskov, 2014; Liu and Mikesell, 2014). This study shows that perception of official corruption can reduce citizens’ subjective wellbeing. Secondly, this study highlights the importance of satisfaction with government performance in wellbeing studies. Government is an important factor which can influence residents’ daily life. Previous studies have tested the effect of government quality, government spending, and government size (Chen et al., 2016; Sequeira et al., 2017; Ott, 2018) on residents’ wellbeing. However, government performance, the outcomes of administrative activities, has been neglected. Our results emphasize that satisfaction with government performance is an important predictor of subjective wellbeing. Thirdly, the results enhance our understanding of the mediating effect of the satisfaction with government performance in the relationship between official corruption and subjective wellbeing. Perception of official corruption can negatively affect subjective wellbeing by reducing residents’ satisfaction with government performance. While previous studies have found the antecedents and outcomes of satisfaction with the government (Van de Walle et al., 2005; Adang and Borm, 2007; Tummers et al., 2016; Salim et al., 2017), our results show the importance of satisfaction with government in the official corruption–citizen wellbeing relationship.

Residents’ high quality of life should be the ultimate government objective (Glaser et al., 2000). However, corrupted officials may focus more on their personal interests rather than on serving the people. Transparency International’s Corruption Perception Index 2019 ranks China the eightieth most corrupt country out of 180.^1^ There are many unique reasons for the corruption of Chinese officials. Firstly, different cultures can influence individuals’ attitude toward the government (Huang, 2018). Confucius culture had embedded in the daily life of Chinese, high power distance, guanxi (connections) and official-orientated thought are the cultural characteristics of Chinese. Residents accept the unequal distribution of power in institutions, and officials prefer to give or receive bribes to get more power. Secondly, with the rapid development of the Chinese economy, businessmen are becoming rich and the price is rising, however, growth of the income of government officials is still slow, so officials tend to be corrupted to get more money. Thirdly, there are still some drawbacks in the design of the supervisory and control system in China. Official corruption facilitates organized crime and flourishes as a consequence of a lack of transparency and weak regulatory practices.

Though there are state anti-corruption agencies in China (e.g., the Central Commission for Discipline Inspection and the Supreme People’s Procuratorate), but the anti-corruption campaign cannot be completed in a short time because of the complexity and differences in corruption. Our findings suggest that satisfaction with government performance can reduce the influence of corruption on happiness. Although corruption can undermine citizens’ happiness, the government can remedy this negative impact through improving government performance. It is therefore necessary to build and strengthen the service-oriented government to satisfy residents’ diverse needs in order to increase their happiness. In addition, the government needs to take measures to increase officials’ income moderately and expand media and citizens’ channels of supervisor to improve government transparency. Furthermore, the problem can be curbed by developing national legislation to fight corruption, putting the power within the confines of the law, and improving the system of sanctions and prevention.

Despite these findings, our research is not without limitations. Firstly, all the findings obtained from this study come from cross-sectional data, which precludes the possibility of making causal statements. In addition, the cross-sectional data were unable to deal with the endogeneity problem presented in the estimation. Meanwhile, the data comes from Chinese sample, so the external validity has not been verified. Future research should therefore use a longitudinal or experimental design to ascertain the causal relationship and avoid the endogeneity problem. And, future research could use large sample under different culture context to verify the generalizability. Secondly, there are different dimensions of wellbeing, and they are all important elements for people’s daily lives. Psychological wellbeing is a stable functional construct associated with adaptive human functioning and positive experiences (Ryff, 1989; Ryan and Deci, 2001). Social wellbeing reflects individuals’ positive social health, including social integration, social contribution, social coherence, social actualization, and social acceptance (Keyes, 1998; Yu et al., 2021). Thus, further research could test different aspects of happiness so as to make the conclusions more accurate and more comprehensive. Thirdly, the Chinese hold different perceptions of government at different levels, and their trust of central government is higher than that of local government (Shi, 2001). Thus, further research could explore perceptions of official corruption and satisfaction with government performance at different levels.

The results obtained from our study by using the data from CGSS 2015 confirm that perception of official corruption has a negative relationship with subjective wellbeing, and satisfaction with government performance is positively related to subjective wellbeing. We further find that satisfaction with government performance serves as a mediator in the relationship between perception of official corruption and subjective wellbeing.

## Data Availability Statement

The raw data supporting the conclusions of this article will be made available by the authors, without undue reservation.

## Ethics Statement

The studies involving human participants were reviewed and approved by Yunnan University of Finance and Economics. Written informed consent for participation was not required for this study in accordance with the national legislation and the institutional requirements.

## Author Contributions

YY and JM designed the research and wrote the manuscript. BG conducted data analysis and verification, modifying and finalizing the paper. All authors contributed to the article and approved the submitted version.

## Funding

This study was supported by the Chinese National Natural Science Fund (72064042;71904070;71763030), the Philosophy and Social Science Research Project in Yunnan Province (QN202026), and the Science Research Foundation for Introduction of Talents of Yunnan University of Finance and Economics (2021D01).

## Conflict of Interest

The authors declare that the research was conducted in the absence of any commercial or financial relationships that could be construed as a potential conflict of interest.

## Publisher’s Note

All claims expressed in this article are solely those of the authors and do not necessarily represent those of their affiliated organizations, or those of the publisher, the editors and the reviewers. Any product that may be evaluated in this article, or claim that may be made by its manufacturer, is not guaranteed or endorsed by the publisher.

## Footnotes

^1^ www.transparency.org
